# Influence of Long Time Storage in Mineral Water on RNA Stability of *Pseudomonas aeruginosa* and *Escherichia coli* after Heat Inactivation

**DOI:** 10.1371/journal.pone.0003443

**Published:** 2008-10-20

**Authors:** Claire Cenciarini, Sophie Courtois, Didier Raoult, Bernard La Scola

**Affiliations:** 1 CIRSEE (Centre International de Recherche Sur l'Eau et l'Environnement) – Suez Environnement, Le Pecq, France; 2 URMITE, CNRS-IRD UMR 6236, Université de la Méditerranée, Faculté de Médecine, Marseille, France; Baylor College of Medicine, United States of America

## Abstract

**Background:**

Research of RNA viability markers was previously studied for many bacterial species. Few and different targets of each species have been checked and motley results can be found in literature. No research has been done about *Pseudomonas aeruginosa* in this way.

**Methodology/Principal Findings:**

Disappearance of 48 transcripts was analyzed by two-steps reverse transcription and real time polymerase chain reaction (RT-PCR) after heat-killing of *Pseudomonas aeruginosa* previously stored in mineral water or not. Differential results were obtained for each target. *16S* rRNA, *23S* rRNA, *groEL*, and *rpmE* were showed as the most persistent transcripts and *rplP*, *rplV*, *rplE* and *rpsD* were showed as the most labile transcripts after *P. aeruginosa* death. However, the labile targets appeared more persistent in bacteria previously stored in mineral water than freshly cultivated (non stored). These nine transcripts were also analyzed in *Escherichia coli* after heat-killing and different to opposite results were obtained, notably for *groEL* which was the most labile transcript of *E. coli*. Moreover, opposite results were obtained between mineral water stored and freshly cultivated *E. coli*.

**Conclusions and Significance:**

This study highlights four potential viability markers for *P. aeruginosa* and four highly persistent transcripts. In a near future, these targets could be associated to develop an efficient viability kit. The present study also suggests that it would be difficult to determine universal RNA viability markers for environmental bacteria, since opposite results were obtained depending on the bacterial species and the physiological conditions.

## Introduction

For many applications, whether food or medical, detection of potentially pathogenic bacteria or only contamination indicators is a necessity. For companies involved in potable water distribution, surveillance of contaminating bacteria, mostly enteric pathogens, represents basis of microbiology quality control. Conventional methods for detection and quantification of these bacteria involve isolation from water filtrates on selective media. These methods typically require days from initiation to readout, and interpretation of results may be difficult because of interfering microflora [1], [2]. Cultivation methods do not detect dead bacteria, which is an advantage. However, viable but not cultivable bacteria, that could be potentially pathogenic, cannot be detected in this way. Different methods for the assessment of bacterial viability have been tested, including cellular integrity, metabolic activities, building of the cellular material, and responsiveness [3]. However, these methods are not specific and these so-called viability markers could stain dead cells for some time after the lethal treatment [4]. By contrast, molecular markers as nucleic acids allow specific detection and quantification of microorganisms. Since real time PCR assays allows now rapid and quantitative detection of DNA from small amounts of bacteria, it could be considered as a possible way to detect water contamination. However, DNA detection may be positive from dead bacteria and does not evaluate bacterial viability [1], [3], [5]–[12]. Thus, DNA detection cannot replace culture-based methods to detect viable bacteria.

An alternative method using rRNA detection is the association of direct viable count (DVC) and fluorescent in situ hybridization (FISH). DVC consists in a revivification step in the presence of a DNA gyrase inhibitor, leading to the cell division inhibition and thus a cell elongation with accumulation of ribosomes. This step is followed by specific 16S rRNA directed fluorescent in situ hybridization. This method allows the specific detection of viable and cultivable and viable but non-cultivable (VBNC) bacteria. DVC-FISH gave good discriminating results for gram-negative bacteria as *E. coli* [13], [14], *H. pylori* [15] or *Enterobacteriaceae* [16], [17] and for gram-positive bacteria [18].

Several studies showed that messenger RNAs could be good candidates for assessment of bacterial viability [1], [8], [19]. The knowledge on the subject remains vague because numerous parameters can modulate the kinetic of mRNA disappearance after bacterial killing. The mostly related parameters are the type of bactericidal treatment (heat, chlorine, UV, Ethanol, drug) and its intensity [1], [6], [9], [10], [20], the post-treatment holding conditions [21], and the physiological state of bacteria before the inactivation treatment [5], [22]. Moreover, different studies disclosed that the decay of various messengers after treatment is heterogeneous: some transcripts persist for a long time [23], [24] while others disappear at once and others put an intermediate time to be completely degraded [10], [25]. Many studies showed that rRNA was detected for very long time (more than 20 to 48 h) after bacterial killing [1], [10], [26]–[28], suggesting that rRNA would not be a good viability marker for the development of a rapid detection method. By contrast, some studies showed that *16S* rRNA disappears relatively rapidly after extreme lethal treatments [26], [29], [30]. Moreover, Aellen *et al.* [20] recently showed that the detection of *16S* rRNA after lethal treatment depended on the choice of the amplified fragment, and *Churruca et al.* [31] showed that 16S rRNA decay depended on the post-treatment holding conditions.

*E. coli* has been the most studied pathogen in the research of RNA targets for viability assessment [1], [9], [10], [21], [29], [32], [33]. However, this bacterium is not an aquatic bacterium but an enteric bacterium that can be isolated in water after faecal contamination. As such, it is a commonly used marker of potable water enteric contamination. Since the goal of our study is to evaluate mRNAs as possible markers of viability for aquatic bacteria, we decided to test *Pseudomonas aeruginosa*. Contaminated water [34] and surfaces in the food industry could become a source of *P. aeruginosa* infections [35], [36]. To our knowledge, no researches of RNA viability markers have been done for this bacterium. In 2007, Matsuda *et al.* [33] suggested that *16S* rRNA could be a viability marker for commensal bacteria, including *P. aeruginosa*, in blood and feces by RT-PCR, but they did not test lethal treatments to confirm this suggestion.

In the aim to find potentially universal viability marker for all waterborne pathogens, we screened messengers encoding the core genes [37] (the minimum set of genes common to all the bacteria), *16S* and *23S* rRNAs and other genes implicated in stress response. However, as some results were contradictory to those previously obtained in literature for *E. coli*, we tested this bacteria in a similar way as a control to check if results obtained for *P. aeruginosa* were really due to a different behavior of transcripts in this bacterium or to experimental conditions. The control herein chosen for viability testing of bacteria was cultivability. We are aware that cultivability is not equivalent to viability. However, we did that choice as it allowed comparison of our results to previously published studies and allows to test the survival of bacteria in a state that is evaluated in commercial water production situations where controls are currently performed by using culture of water filtrates.

## Results

### Inactivation of *E. coli* and *P. aeruginosa* cells by heat treatment at 65°C during 30 minutes

From positive controls of *P. aeruginosa* spiked water and *E. coli* spiked water, 10^6^ to 10^7^ CFU/ml were quantified by colony count. From each heat-treated samples at 65°C for 30 min, no colony grew, neither on blood agar plates incubated for 48 h, nor on R2A agar plates incubated for 1 week, showing the effectiveness of the inactivation treatment.

In parallel, the size RNA profile before and following heat lethal-treatment of *P. aeruginosa* was checked by bioanalyzis (figure S1 of supplementary data). The positive controls gave a standard profile, with expected *16S* and *23S* rRNA picks, and heat-killed cells gave a highly degraded but persistent profile immediately and 24 hours after treatment. Similar results were obtained with *E. coli* (data not shown).

Given their unculturability and their highly degraded RNA profile, we considered that 65°C 30 min heat-treated populations were well inactivated.

### Heterogeneous behavior of tested transcripts after heat-treatment of *P. aeruginosa*

**Figure 1:**
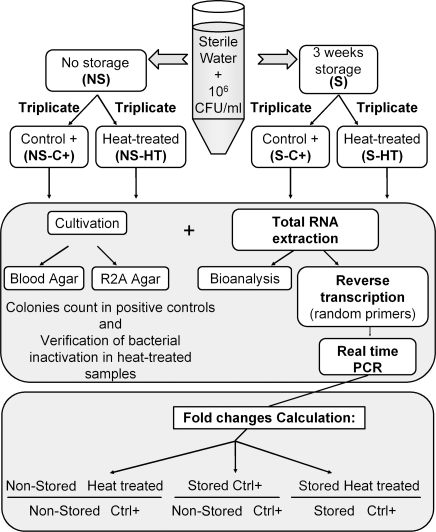
Study design.

48 transcripts corresponding to core genes plus *spoT*, *sodB* and *groEL* mRNAs, and ribosomal rRNAs were analyzed by real-time RT-PCR before (for positive control), immediately after and 24 hours after heat killing. Results were obtained from 3 aliquots proceeded in the same time (Figure 1, study design). According to total RNA profile observations, the real-time RT-PCR analysis showed that amounts of all of transcripts started to decrease immediately after heat treatment. Different levels of persistence, with fold-changes of 1.7×10^−1^ (or −0.78 log_10_) still 3.0×10^−3^ (or −2.52 log_10_), were observed immediately after bacterial heat-inactivation. As expected, ribosomal RNAs were among the most persistent transcripts (see supplementary data, figure S2.A).

The general tendency was confirmed and strengthened 24 hours after the treatment, with decrease levels of −1.28 log_10_ to −3.19 log_10_ (supplementary data, figure S2.B). Surprisingly, none of tested transcripts totally disappeared 24 hours after heat treatment whereas the inactivated population was stored at ambient temperature. Finally, after testing these 48 transcripts, 12 could be considered as labile transcripts with a minimum fold decrease of −2.65 log_10_ (value arbitrary chosen), and 11 of them could be considered as persistent transcripts with a maximum fold decrease of −1.75 log_10_. Based on reproducibility, and after appreciation of the initial Ct, few transcripts were selected for the next step. *rplP*, *rplV*, *rplE* and *rpsD* mRNAs were selected among the labile transcript, and *obg*, *groEL* and *rpmE* mRNAs and *16S* and *23S* rRNAs were selected among the persistent transcripts.

To confirm these results with more specific analysis, specific TaqMan labeled probes and new primer pairs were designed to restart the real-time PCR analyze for the nine selected targets. Results were in accordance (Table 1), excepted for *obg* mRNA. From labile transcripts, 24 h after lethal heat-treatment the average fold decrease was of −2.83 log_10_, the most labile being *rplP* mRNA. From persistent transcripts, the average fold decrease was of −1.13 log_10_ and *groEL* mRNA appeared more persistent than *23S* rRNA by using SYBR green.

**Table 1:** Decrease levels of selected transcripts after bacterial death.

Transcript	Fold change(a) (log_10_)			
	*P. aeruginosa*		*E. coli*	
	Non stored	3 weeks stored	Non stored	3 weeks stored
**rplP**	−3.23	−2.19	−2.88	−1.79
**rplV**	−2.84	−1.88	−2.79	−1.16
**rplE**	−2.80	−1.93	−2.07	−1.74
**rpsD**	−2.62	−1.56	−2.73	−2.16
**Obg**	−2.28	−1.09	−2.58	−2.76
**16S rRNA**	−1.42	−1.42	−1.66	−1.92
**groEL**	−0.79	−1.04	−3.09	−3.12
**rpmE**	−1.31	−0.70	−2.20	−2.52
**23S rRNA**	−1.29	−0.77	−0.64	−1.57

(a) F.C. = 2^−(Ct target treated− Ct target Ctrl +)^.

Ratios calculated between fold change of the most labile and the most persistent transcripts are showed in Table 2. The best ratio was obtained for *groEL*/*rplP* ( = 276).

**Table 2:** Ratios between decrease levels of persistent and labile transcripts.

Transcript	*P. aeruginosa*						*E. coli*		
	Non stored			3 weeks stored			3 weeks stored		
	23S/target	GroEL/target	rpmE/target	23S/target	GroEL/target	rpmE/target	23S/target	rplE/target	16S/target
**rplP**	87	276	84	20	15	18			
**rplV**	35	113	34	14	10	12			
**rplE**	32	102	31	14	11	12			
**rpsD**	21	68	21	5	4	4			
**groEL**							42	24	16

### Effect of long time storage in mineral water before lethal heat-treatment of *P. aeruginosa* on 9 selected transcripts behavior

As for samples freshly cultivated, 10^6^ to 10^7^ CFU/ml were quantified in samples of *P. aeruginosa* stored 3 weeks in mineral water, indicating that there was no increase or decrease of the population after storage. After heat-treatment at 65°C for 30 minutes of these samples stored in mineral water, no colony grew on blood agar plates or on R2A agar plates, indicating that bacterial population was inactivated by the treatment.

Bioanalyzis of total RNA profile from positive control and heat-treated samples of previously mineral water stored population (complementary data) were similar to these obtained from freshly cultivated *P. aeruginosa*.

**Figure 2:**
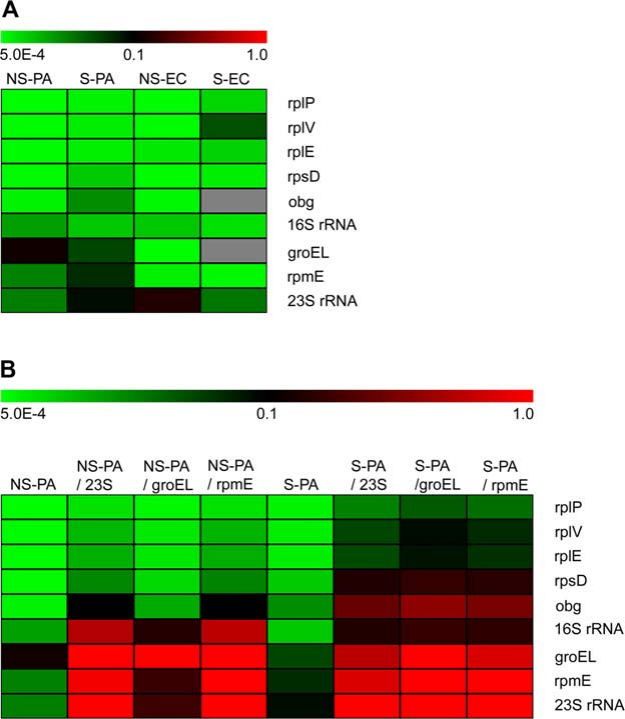
Real time RT-PCR results analyzed by using TMev software. 24 hours after heat-killing, RNA extraction and random reverse transcription, the transcripts were analyzed by real time PCR and fold changes were calculated between T 0h positive controls and heat-treated samples. Fold changes results were analyzed by TMev software. A) Comparison transcripts decay profile 24 hours after lethal heat-treatment of *P. aeruginosa* (PA) and *E. coli* (EC) in non-stored (NS) and previously 3 weeks stored in mineral water (S) conditions. B) Comparison of results analysis of *P. aeruginosa* with or without calculation of ratios with one of the most persistent transcript Ct value. NSPA = Non-stored *P. aeruginosa*; SPA = Stored *P. aeruginosa*; NSEC = Non-stored *E. coli*; SEC = Stored *E. coli*.

Real time RT-PCR hybridization probes results showed that from mineral water stored *P. aeruginosa* (column 1 and 2 of Figure 2.A and Table 1), *rpsD* mRNA and *16S* rRNA diverged from their respective groups, with intermediate decrease levels. The labile group conserved *rplP*, *rplV* and *rplE* as the most labile transcripts, with an average fold decrease of −2.21 log_10_ 24 h after lethal heat-treatment; The persistent group conserved *obg*, *groEL*, *rpmE* and *23S* rRNA with an average fold decrease of −1.13 log_10_. As showed in Table 2, the best ratio obtained between fold changes of labile and persistent transcripts on mineral water stored bacteria was obtained with *23S* rRNA/*rplP* with a value of 20.

### Comparison between *E. coli* and *P. aeruginosa*

Real time RT-PCR, using SYBR green technology, were proceeded by using *E. coli* specific primers for the nine transcripts selected for *P. aeruginosa*. 24 hours after treatment, fold changes were calculated between heat-killed samples and positive controls for each transcript, as calculated above for *P. aeruginosa*. From the fresh *E. coli* population (column 3 of Figure 2.A and Table 1), *rplP*, *rplV* and *rpsD* mRNAs were among the labile transcripts, as for *P. aeruginosa*, but *rplE* showed an intermediate decrease level. In the persistent transcripts group, *16S* rRNA was also one of the most persistent transcripts in freshly cultivated *E. coli* population, but *rpmE* mRNA showed an intermediate level of decrease and *groEL* mRNA was the less persistent from freshly cultivated *E. coli* in contrast to *P. aeruginosa*. Results obtained from mineral water stored *E. coli* population (column 4 of Figure 2.A and Table 1) showed stronger differences compared to results presented above for *P. aeruginosa* and looked different from those obtained from fresh population. *RplV* mRNA appeared as the most persistent transcripts, whereas *groEL*, *rpmE* and *obg* became the most labile.

## Discussion

Results obtained in this study showed that among 48 transcripts analyzed form heat-killed *P. aeruginosa*, 2 groups could be classified in persistent transcripts (*23S* and *16S* rRNA, *rmpE*, *groEL* and *obg* mRNAs) and labile transcripts (*rplP*, *rplV*, *rplE* and *rpsD* mRNA). These observations appeared reliable as they were triplicated and confirmed when tested by using TaqMan technology. Transcripts of these genes were also analyzed for *E. coli*, and results were verified by using both SYBR green and TaqMan technologies on triplicates.

This work shows that the RNA disappearance after bacterial death is not uniform, as previously reported results [1], [10], [38]. We also confirmed that *16S* and *23S* ribosomal RNAs were among the most persistent transcripts [1], [10], [26]–[28]. However, we found that some transcripts could be even more persistent. These observations suggest that general kinetic of transcripts decay after-death is not predictable by leaning on the analysis of only few transcripts. It is necessary to study the correlation between cell mortality and disappearance of each tested transcript before to use it for viability assessment.

This study also suggests the considerable role played by the physiological condition of the population before lethal treatment. For *P. aeruginosa*, we observed that differences in the behavior of the labile group and the persistent group of transcripts were lower in mineral water stored bacteria than in freshly cultivated bacteria. For *E. coli*, we obtained even stronger differences between the two physiological conditions as opposite results were obtained for *rplV*. These results support those of Coutard *et al.* [5] who showed differences in the persistence of *rpoS* after heat killing freshly cultivated or viable but non cultivable *Vibrio parahaemolyticus*.

We observed differences in the transcript decrease between *E. coli* RNAs and *P. aeruginosa* RNAs, except for ribosomal RNAs. The most different was *groEL* mRNA. In *P. aeruginosa*, *groEL* mRNA was one of the most persistent transcript, as for *V. cholerae* [25]. However, this mRNA was the most labile transcript in *E. coli* in our work and this of Sheridan *et al.* [1]. Such difference in this transcript persistence in two different bacteria was unexpected as *groEL* is a key for cell survival [39], [40] and as it plays an major role against thermal shock of 45 to 55°C or stress [39]–[42].

The current criteria for discrimination between viable and dead bacteria is the RNA level ratio before and after killing cells [3]. However, the results obtained in our study highlight how difficult it is to establish a clear correlation between viability and transcripts in *P. aeruginosa* as none of the tested transcripts completely disappeared. However, *rplP*, *rplV*, *rplE* and *rpsD* can be selected as the best viability markers. In *E. coli*, only *groEL* mRNA showed a complete disappearance. Moreover, in this study we found that the physiological conditions (freshly cultivated or long time mineral water stored cells) influenced the transcription profile. This study showed that it will be difficult to determine universal RNA viability markers for environmental bacteria, since opposite results were obtained from *E. coli* and *P. aeruginosa*. Moreover, other tests will have to be done to complement culturability testing by viability testing [43] to ensure that bacteria are efficiently killed. Studies performed by using microarrays for each bacterial species, one by one, with a large number of targets, testing different physiological conditions may allow defining optimal targets for this purpose.

## Materials and Methods

### Study design

Figure 1 represents the study design. Commercialized natural mineral water (pH 7.2; mineral content [in mg liter−1]: Na+, 5; K+, 1; Ca2+, 78, Mg2+ 24; Cl−, 4.5; SO42− 10; NO3−, 3.8; HCO3−, 357) was sterilized by filtration on 0.22 µm pore size membrane and spiked with an average of 10^6^ CFU/ml of freshly cultivated *P. aeruginosa* or *E. coli* cells. One part of the spiked water was stored for 3 week, and the other part (freshly cultivated population) was used immediately for heat treatment. To favor the temperature exchange between the dry bath and the samples, 1.2 ml aliquots were prepared and triplicates of aliquots were proceeded for each condition. Treated aliquots were heated at 65°C during 30 minutes and positive controls were kept at room temperature. Each aliquot was then fast cooled on ice and kept in the dark at room temperature still analysis. Immediately and 24 hours after, 100 µl aliquot were used for plating on blood agar and R2A agar and 1 ml was used for total RNA extraction. The total RNA profile size was analyzed, and each transcript was analyzed by two steps real time RT-PCR. Fold changes were then calculated for different transcripts to evaluate their decrease level between heat-killed samples and positive controls (non-heat treated).

In the aim to work in physiological condition, closer to this met in environmental water, this experiment and analysis was exactly reproduced with the same spiked water stored during 3 weeks. Fold changes were calculated for each transcripts between 3 weeks stored and heat-killed samples and 3 weeks stored positive controls (non heat-killed).

### Bacterial strains and growth conditions

*Pseudomonas aeruginosa* CIP 100720 and *Escherichia coli* CIP 106878 were used in this study. Bacterial suspensions were prepared in 10 ml of Liquid Luria-Bertani (LB) broth and incubated over night on a shaker at 30°C and 37°C for *P. aeruginosa* and *E. coli* respectively. Colony forming units (CFU) were counted after plating 100 µl of samples on sheep blood agar (COS; BioMérieux, Marcy l'Etoile, France) and incubation for 24 to 48 hours at 37°C and after plating on R2A agar (Becton Dickinson, Heidelberg, Germany) and incubation for 1 week at 22 to 25°C.

### Primers and probes

The function of each analyzed gene is presented in Table 3. Primers and probes were designed by using Primer 3 [44] and specificity was verified with BLASTN program. Sequences of primers and probes, used concentrations in PCR and annealing temperature are presented in Table S1 of the supplementary data.

**Table 3:** Genes analyzed in this study and corresponding function.

Gene	Fonction	COG Category
16S rRNA	Ribosomal RNA	/
23S rRNA	Ribosomal RNA	/
ftsE	Predicted ATPase involved in cell division	D: Cell cycle control, cell division, chromosome partitioning
adk	Adenylate kinase	F: Nucleotide transport and metabolism
efp	Translation elongation factor P	J: Translation, ribosomal structure and biogenesis
frr	Ribosome recycling factor	J: Translation, ribosomal structure and biogenesis
fusA	Translation elongation factors (GTPases)	J: Translation, ribosomal structure and biogenesis
glnS	Glutamyl- and glutaminyl-tRNA synthetases	J: Translation, ribosomal structure and biogenesis
ileS	Isoleucyl-tRNA synthetase	J: Translation, ribosomal structure and biogenesis
infB	Translation initiation factor 2 (IF-2; GTPase)	J: Translation, ribosomal structure and biogenesis
infC	Translation initiation factor 3 (IF-3)	J: Translation, ribosomal structure and biogenesis
leuS	Leucyl-tRNA synthetase	J: Translation, ribosomal structure and biogenesis
prfA	Protein chain release factor A	J: Translation, ribosomal structure and biogenesis
prfB	Protein chain release factor B	J: Translation, ribosomal structure and biogenesis
rplA	Ribosomal protein L1	J: Translation, ribosomal structure and biogenesis
rplB	Ribosomal protein L2	J: Translation, ribosomal structure and biogenesis
rplC	Ribosomal protein L3	J: Translation, ribosomal structure and biogenesis
rplE	Ribosomal protein L5	J: Translation, ribosomal structure and biogenesis
rplK	Ribosomal protein L11	J: Translation, ribosomal structure and biogenesis
rplL	Ribosomal protein L7/L12	J: Translation, ribosomal structure and biogenesis
rplM	Ribosomal protein L13	J: Translation, ribosomal structure and biogenesis
rplN	Ribosomal protein L14	J: Translation, ribosomal structure and biogenesis
rplO	Ribosomal protein L15	J: Translation, ribosomal structure and biogenesis
rplP	Ribosomal protein L16/L10E	J: Translation, ribosomal structure and biogenesis
rplQ	Ribosomal protein L17	J: Translation, ribosomal structure and biogenesis
rplR	Ribosomal protein L18	J: Translation, ribosomal structure and biogenesis
rplS	Ribosomal protein L19	J: Translation, ribosomal structure and biogenesis
rplV	Ribosomal protein L22	J: Translation, ribosomal structure and biogenesis
rpmE	Ribosomal protein L31	J: Translation, ribosomal structure and biogenesis
rpsC	Ribosomal protein S3	J: Translation, ribosomal structure and biogenesis
rpsD	Ribosomal protein S4	J: Translation, ribosomal structure and biogenesis
rpsE	Ribosomal protein S5	J: Translation, ribosomal structure and biogenesis
rpsG	Ribosomal protein S7	J: Translation, ribosomal structure and biogenesis
rpsH	Ribosomal protein S8	J: Translation, ribosomal structure and biogenesis
rpsI	Ribosomal protein S9	J: Translation, ribosomal structure and biogenesis
rpsJ	Ribosomal protein S10	J: Translation, ribosomal structure and biogenesis
rpsL	Ribosomal protein S12	J: Translation, ribosomal structure and biogenesis
rpsN	Ribosomal protein S14	J: Translation, ribosomal structure and biogenesis
rpsP	Ribosomal protein S16	J: Translation, ribosomal structure and biogenesis
rpsQ	Ribosomal protein S17	J: Translation, ribosomal structure and biogenesis
rpsR	Ribosomal protein S18	J: Translation, ribosomal structure and biogenesis
trmD	tRNA-(guanine-N1)-methyltransferase	J: Translation, ribosomal structure and biogenesis
tsf	Translation elongation factor	J: Translation, ribosomal structure and biogenesis
tufB	GTPases - translation elongation factors	J: Translation, ribosomal structure and biogenesis
rpoB	DNA-directed RNA polymerase, beta subunit/140 kD subunit	K: Transcription
lepA	Membrane GTPase LepA	M: Cell wall/membrane/envelope biogenesis
gyrB	Type IIA topoisomerase (DNA gyrase/topo II, topoisomerase IV), B subunit	N: Cell motility
groEL	Chaperonin GroEL (HSP60 family)	O: Posttranslational modification, protein turnover, chaperones
hflB	ATP-dependent Zn proteases	O: Posttranslational modification, protein turnover, chaperones
sodB	Superoxide dismutase	P: Inorganic ion transport and metabolism
obg	Predicted GTPase	R: General function prediction only
spoT	Guanosine polyphosphate pyrophosphohydrolases/synthetases	TK: Signal transduction mechanisms+Transcription

### Heat treatment of bacteria spiked in water samples

Sterile water was spiked with freshly cultivated *P. aeruginosa* or *E. coli* previously washed with physiological water and with sterile water to a final concentration of 10^6^ to 10^7^ CFU/ml. 1.2 ml Aliquots were prepared in 1.5 ml Eppendorf tubes and incubated at 65°C during 30 minutes in a dry bath, or kept at ambient temperature for positive controls. Aliquots were then quickly cooled on ice for 2 minutes and kept at room temperature still plating and RNA extraction, immediately and 24 hours after heat treatment. For verifying the inactivation treatment efficiency, 100 µl of each sample were plated, after serial dilutions for positive controls, on blood agar and 100 µl were plated on R2A agar. R2A medium, associated with reduced incubation temperatures (20 to 30°C) for a period of at least seven days, yields the highest total bacterial numbers in an evaluation of waterborne bacteria than did using an enriched medium as blood agar or trypticase soy agar [45]–[47]. The R2A agar is then considered as the gold standard for measuring heterotrophic bacteria in water [48].

### Bacterial storage in mineral water

The spiked water was incubated in glass flasks at 4°C in the dark for 3 weeks, without addition of nutriments. This treatment intended to reproduce starvation conditions as it was supposed to evaluate survival of bacteria in a state that could be encountered in commercial water production testing. However, we did not use the term “starvation” as bacteria maintained in mineral water do not die quickly as observed in dematerialized water [49]–[51].

### RNA isolation and purification, and elimination of contaminating DNA

The pellet of the 1 ml remaining of each aliquot was first lyzed by incubation with 100 µl of TE containing 600 µg/ml of lysozyme, during 5 to 10 minutes. Total RNA extraction and purification from samples was then proceeded by using RNeasy MiniKit (Qiagen, Courtaboeuf, France) according to manufacturer's instructions. RNA samples were eluted in 40 µL of RNase Free water. To ensure a complete elimination of contaminating DNA, two DNase treatments were applied on RNA samples. The first treatment was done by using RNase-Free DNase I (Qiagen, Courtaboeuf, France) directly applied on the RNeasy column during 15 minutes at room temperature, according to the manufacturer's instructions. The second digestion was done by using the RNase-Free RQ1 DNase (Promega, Charbonnières-les-Bains, France). According to manufacturer's instructions, 1U of DNase and 1 µl of DNase 10× Reaction Buffer were added in 8 µl of RNA sample and incubated 30 minutes at 37°C. The reaction was stopped by addition of 1 µl of the DNase stop solution and incubation 10 minutes at 65°C.

### Analysis of total RNA size profile

Profile size of purified RNA from samples was evaluated on an Agilent 2100 Bioanalyzer instrument by using the RNA 6000 Pico LabChip kit (Agilent Technologies, Massy, France). 1 µl of each sample was analyzed out according to the manufacturer's protocol. Although the Bioanalyzer is not considered as a quantification tool, it allows for extensive RNA quality evaluation including identification of degraded RNA, rRNA/mRNA-fractions and DNA contamination [52], [53], and the using of PicoChips allow a very sensitive detection.

### Reverse transcription and real time PCR

cDNA were synthesized by using the M-MLV reverse transcriptase (Invitrogen, Cergy Pontoise, France.) according to the manufacturer's instructions. Briefly, 5 µl of a total volume of 40 µl of extracted RNA was reverse transcribed in a reaction volume of 20 µl containing dNTPs, random primers, DTT, 5× buffer and RNase Out. The reaction mixtures were incubated in a 2720 thermalCycler (Applied Biosystems, Courtaboeuf, France) at 37°C for 50 minutes, and heating at 95°C for 5 min terminated the reaction.

Specific primers and probes were designed by using the Primer3 program [44], Source code available at http://fokker.wi.mit.edu/primer3/) from DNA sequences, submitted to the EMBL/GenBank databases. 18 to 20 bp Primers were selected to amplify 90 to 180 bp fragment size and synthesized by Eurogentec (Angers, France). For selected genes, 25 to 30 bp TaqMan probes were designed to have an annealing temperature 10°C upper to primers annealing temperature. These probes were synthesized and labeled on 5′ extremity with FAM as fluorochrome and on 3′ extremity with TARMA as quencher by Operon (Cologne, Germany). PCR conditions were optimized for each primer pairs and probes by modifying annealing temperature and final concentration to avoid primer dimers and unspecific amplifications. Table S1 (supplementary data) shows primers and probes sequences, melting temperatures and used concentrations for the real-time PCR.

Real time PCRs were performed in a Light Cycler 2.0 (Roche) for *P. aeruginosa* analysis, and in a SMART Cycler II (Cepheid, Maurens-Scopont, France) for *E. coli* analysis, which allows performing different amplification in a unique run, that was less time consuming. Analysis with SYBR green technology were realized by using the LightCycler FastStart DNA Master Mix SYBR Green I kit (Roche Diagnostics, Meylan, France). Amplification was done by using the following program: 10 min – 95°C for activation of the enzyme, 40×[95°C – 10 sec; X°C – 4 sec (see Table S1 in supplementary data); 72°C – 5 sec] for amplification, and [95°C – 0 sec; 65°C – 15 sec, increased to 95°C by 0.1°C/sec] for melting curves analysis. TaqMan analysis were realized by using the FastStart DNA Master Hybridization Probes kit (Roche Diagnostics), with the following amplification program: 10 min – 95°C for activation of the enzyme, 40×[95°C – 10 sec; 60°C – 10 sec; 72°C – 10 sec]. Before *E. coli* analysis, the Taq polymerase was treated by RQ1 DNase (Promega, France) because of an *E. coli* DNA contamination of the enzyme. Every PCRs were done with 2 µl of cDNA in a final volume of 20 µl. Controls containing not reverse transcripted RNA, water extracted sample, and pure water instead of sample were done systematically for each target.

### Results analysis

Results were analyzed by determining a “fold-change” of transcripts amplification between dead cells and positive controls. Usually, in transciptome analysis, the fold-change is calculated by using the following conventional mathematical formula [54]:
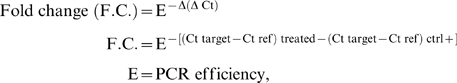
“ref” is usually a house keeping gene that relate the quantity of total live cells in the sample.

The aim of the present study was to investigate the RNA decay in dead bacteria compared to live bacteria. In dead cells, RNA corresponding to house keeping genes should be also degraded, and could not relate the total number of bacteria, including live plus dead cells, and this number remained theoretically unchanged between the positive control and the treated sample. Then, we admitted that the number of cells could constitute the “ref”. If Ct_ref_ treated = Ct_ref_ ctrl, the previous formula became:
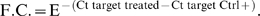



Given the important number of analyzed genes, internal standard curve was not proceeded for each real time PCR. However, the good PCR efficiency was previously verified by external standard curves with different primers concentrations and annealing temperatures for each primer pairs before using. In general, it is considered that E = 2. Then, results were interpreted by using the following formula:
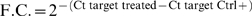



## Supporting Information

Figure S1 (3.37 MB DOC)

Figure S2 (1.29 MB DOC)

Table S1 Oligonucleotide primer and probe sequences used for real time PCR in this study
(0.29 MB DOC)
