# Supplementary figures and images for: Influence of Long Time Storage in Mineral Water on RNA Stability of Pseudomonas aeruginosa and Escherichia coli after Heat Inactivation
Source: PLoS One. 2008 Oct 20;3(10):e3443. doi: 10.1371/journal.pone.0003443 (PMC2566809; doi:10.1371/journal.pone.0003443)

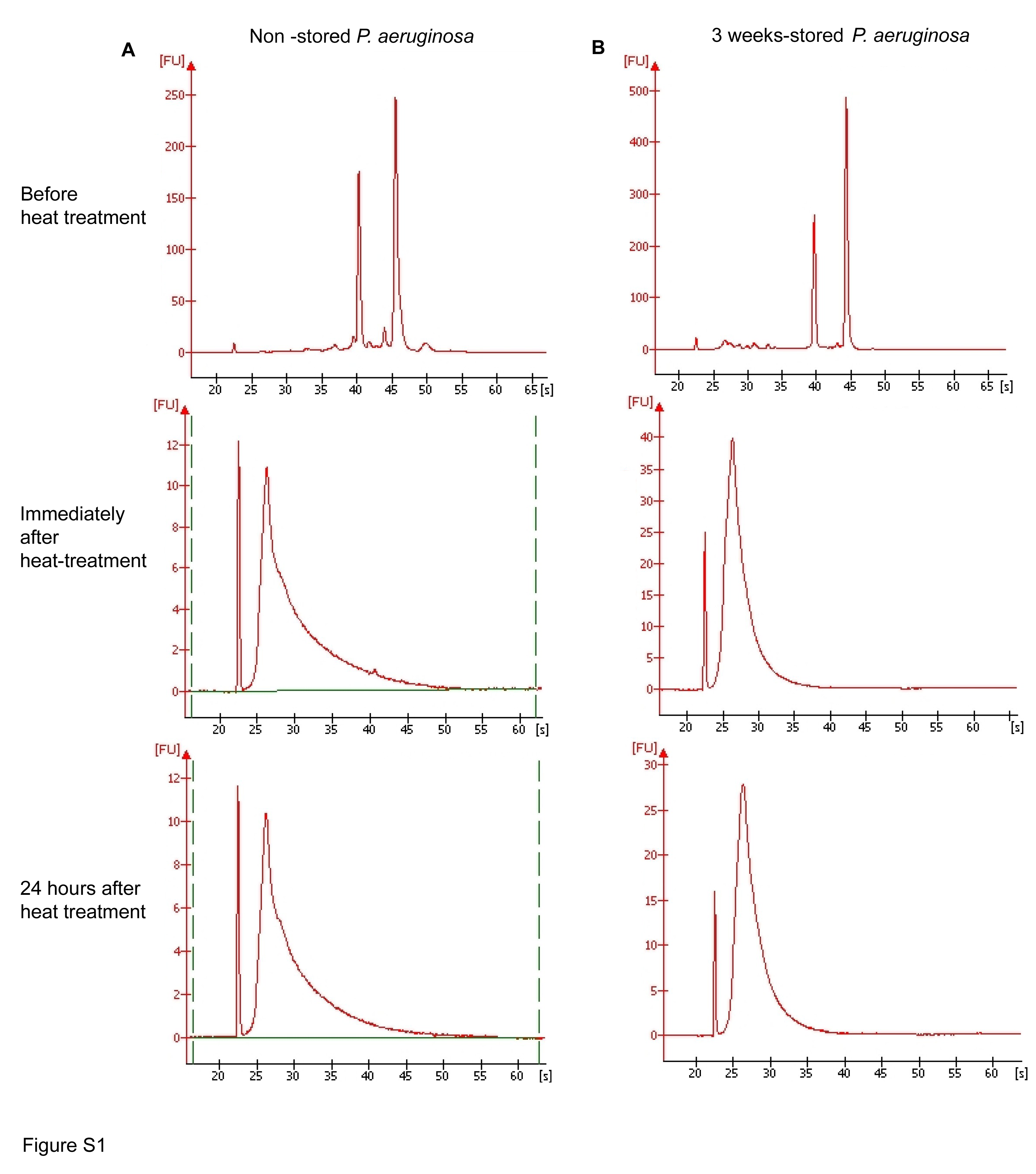

Supplement: Figure S1 — (3.37 MB DOC) [file pone.0003443.s001.doc]

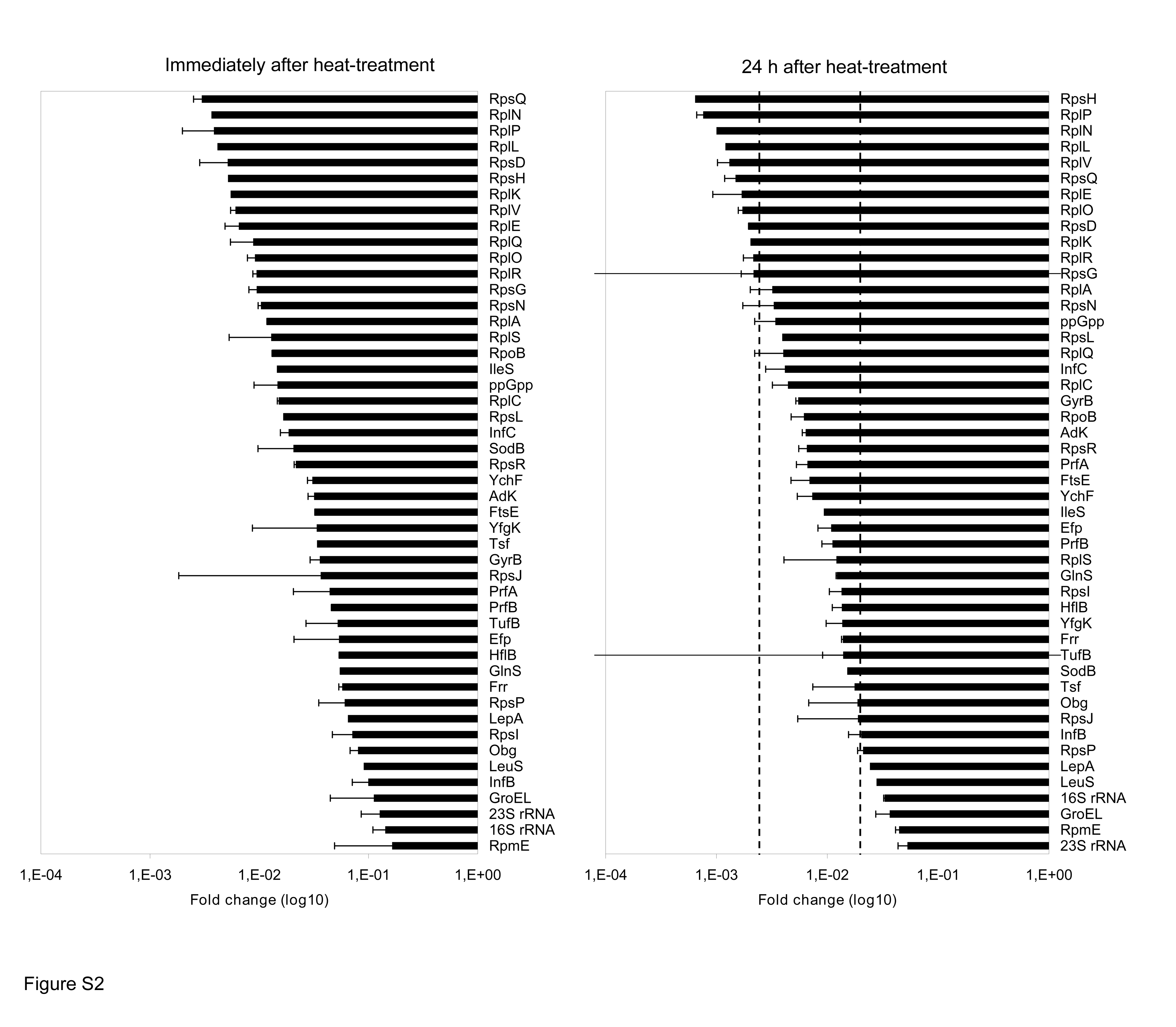

Supplement: Figure S2 — (1.29 MB DOC) [file pone.0003443.s002.doc]
