# Supplementary material for: Influence of Long Time Storage in Mineral Water on RNA Stability of Pseudomonas aeruginosa and Escherichia coli after Heat Inactivation
Source: PLoS One. 2008 Oct 20;3(10):e3443. doi: 10.1371/journal.pone.0003443 (PMC2566809; doi:10.1371/journal.pone.0003443)
Supplement: Table S1 — Oligonucleotide primer and probe sequences used for real time PCR in this study (0.29 MB DOC) [file pone.0003443.s003.doc]

| **Bacteria** | **Target sequence** | **Primer** | **Sequence (5' - 3')** | **Amplicon length** | **Annealing temp.** | **Concentration^a^** |
| --- | --- | --- | --- | --- | --- | --- |
| *P. aeruginosa* | adk | Forward / SYBR | CGAGGACGACAAGGAAGAGA | 128 | 60 °C | 0.3 µM |
|  |  | Reverse / SYBR | CGGCGATGCTGTGGTATT |  |  | 0.3 µM |
|  |  |  |  |  |  |  |
| *P. aeruginosa* | efp | Forward / SYBR | CATCTGCGAAGCCGTGTTCTAC | 129 | 62°C | 0.5 µM |
|  |  | Reverse / SYBR | GCGGTCTTCATCACCTTGCC |  |  | 0.5 µM |
|  |  |  |  |  |  |  |
| *P. aeruginosa* | frr | Forward / SYBR | ATCCTGGATAGCGTGATGGTTTC | 125 | 62°C | 0.5 µM |
|  |  | Reverse / SYBR | GCCTGGATCATGCTCTTGTCG |  |  | 0.5 µM |
|  |  |  |  |  |  |  |
| *P. aeruginosa* | ftsE | Forward / SYBR | AGGTCGGCAAACGCTATCCC | 149 | 62°C | 0.5 µM |
|  |  | Reverse / SYBR | GGTCGGTCGCTCCATCGC |  |  | 0.5 µM |
|  |  |  |  |  |  |  |
| *P. aeruginosa* | fusA2 | Forward / SYBR | GTATCTGGAAGGCGAGGAACTGAG | 105 | 62°C | 0.2 µM |
|  |  | Reverse / SYBR | TTGAACGAGGAGCCGAGCAC |  |  | 0.2 µM |
|  |  |  |  |  |  |  |
| *P. aeruginosa* | glnS | Forward / SYBR | GTCCGCTCTACGAGTGGTTCC | 96 | 62°C | 0.5 µM |
|  |  | Reverse / SYBR | TGGTCACGGTGTAGTTCAGGTTG |  |  | 0.5 µM |
|  |  |  |  |  |  |  |
| *P. aeruginosa* | groEL | Forward / SYBR | CGGCTTCAACGCTGCTAC | 151 | 62°C | 0.5 µM |
|  |  | Reverse / SYBR | TTCCACGATCTCGGCAAC |  |  | 0.5 µM |
|  |  |  |  |  |  |  |
| *P. aeruginosa* | groEL | Forward / Probe | CCAGGTAGGCACCATCTCC | 89 | 60°C | 0.5 µM |
|  |  | Reverse / Probe | TCACGCCTTCTTTACCGACT |  |  | 0.5 µM |
|  |  | Probe | CAGATCATTGCCGAAGCCATGGAAA |  |  | 0.2 µM |
|  |  |  |  |  |  |  |
| *P. aeruginosa* | gyrB | Forward / SYBR | AACAAGGTCTGGGAACAGGTCTAC | 136 | 62°C | 0.5 µM |
|  |  | Reverse / SYBR | AACTGAAGTGGATGTTGCTGAAGG |  |  | 0.5 µM |
|  |  |  |  |  |  |  |
| *P. aeruginosa* | hflB | Forward / SYBR | CGAGTGACCGTGGATGGCTATG | 149 | 62°C | 0.5 µM |
|  |  | Reverse / SYBR | TGCTGTTCGGGCTGCTTGC |  |  | 0.5 µM |
|  |  |  |  |  |  |  |
| *P. aeruginosa* | ileS | Forward / SYBR | TACGGGATGAGCAACGACGAC | 90 | 62°C | 0.2 µM |
|  |  | Reverse / SYBR | GATGAACTGACCGCCGAAGAAC |  |  | 0.2 µM |
|  |  |  |  |  |  |  |
| *P. aeruginosa* | infB | Forward / SYBR | CCTGCCGCCGTGGAAGAG | 97 | 60 °C | 0.3 µM |
|  |  | Reverse / SYBR | GGTGCTGGGTGTGCTTGC |  |  | 0.3 µM |
|  |  |  |  |  |  |  |
| *P. aeruginosa* | infC | Forward / SYBR | TCGTGAGGTACGGTTGATTG | 96 | 60 °C | 0.3 µM |
|  |  | Reverse / SYBR | AGGTCCAGCTTCGCCTCTT |  |  | 0.3 µM |
|  |  |  |  |  |  |  |
| *P. aeruginosa* | lepA | Forward / SYBR | CGGGTGAAGAAGGGCGACAAG | 92 | 62°C | 0.3 µM |
|  |  | Reverse / SYBR | TCGGTGTGCTTCGGAGTGAATAC |  |  | 0.3 µM |
|  |  |  |  |  |  |  |
| *P. aeruginosa* | leuS | Forward / SYBR | GCACGAGCAATATCAGCCACTC | 104 | 62°C | 0.3 µM |
|  |  | Reverse / SYBR | AGAACTTCTCCTTGTCGGGTAGC |  |  | 0.3 µM |
|  |  |  |  |  |  |  |
| *P. aeruginosa* | obg | Forward / SYBR | CAGTGTCGGTCGCTACAAGAG | 142 | 62°C | 0.5 µM |
|  |  | Reverse / SYBR | CGCCATGTCCACCAGATGC |  |  | 0.5 µM |
|  |  |  |  |  |  |  |
| *P. aeruginosa* | obg | Forward / Probe | AAGCTGGAACTGAAGGTGCT | 126 | 60°C | 0.5 µM |
|  |  | Reverse / Probe | CAGGGTGGTGAACGGATAGT |  |  | 0.5 µM |
|  |  | Probe | CAAGAGCACTTTCATCCGTGCAGTGTC |  |  | 0.2 µM |
|  |  |  |  |  |  |  |
| *P. aeruginosa* | spoT | Forward / SYBR | AGGAGTCGCTGGTCAACTG | 148 | 62°C | 0.5 µM |
|  |  | Reverse / SYBR | ATGATGCGGAAGGCGTAG |  |  | 0.5 µM |
|  |  |  |  |  |  |  |
| *P. aeruginosa* | prfA | Forward / SYBR | CGACCTGTTCCGCATGTATT | 139 | 62°C | 0.3 µM |
|  |  | Reverse / SYBR | GAGCTTGGCGTAGACGTTGT |  |  | 0.3 µM |
|  |  |  |  |  |  |  |
| *P. aeruginosa* | prfB | Forward / SYBR | GAAATCAACCCGATCCTCAA | 99 | 62°C | 0.3 µM |
|  |  | Reverse / SYBR | ACCAGGCGATCATGCTTCT |  |  | 0.3 µM |
|  |  |  |  |  |  |  |
| *P. aeruginosa* | rplA | Forward / SYBR | GCGATTGCCGAGAAAGTAG | 90 | 62°C | 0.3 µM |
|  |  | Reverse / SYBR | CTTGAACTTGATGGTGGACAG |  |  | 0.3 µM |
|  |  |  |  |  |  |  |
| *P. aeruginosa* | rplB | Forward / SYBR | ACATCGCTCTGCTGAAGTATGC | 119 | 62°C | 0.3 µM |
|  |  | Reverse / SYBR | GCTGTTGCCTGCCTTGATCG |  |  | 0.3 µM |
|  |  |  |  |  |  |  |
| *P. aeruginosa* | rplC | Forward / SYBR | GCAGTATGCCGCTGGTGACC | 91 | 62°C | 0.5 µM |
|  |  | Reverse / SYBR | GAAGCCCTTACCCTTGGACTCG |  |  | 0.5 µM |
|  |  |  |  |  |  |  |
| *P. aeruginosa* | rplE | Forward / SYBR | GTAAGTCCATCGCAGGCTTCAAG | 102 | 62°C | 0.3 µM |
|  |  | Reverse / SYBR | ACAGCAGACGATCCAGGAACTC |  |  | 0.3 µM |
|  |  |  |  |  |  |  |
| *P. aeruginosa* | rplK | Forward / SYBR | ACCGTTTACAGCGACCGTAGC | 117 | 62°C | 0.2 µM |
|  |  | Reverse / SYBR | GACTTTCTGGGAGTTCGGACGAG |  |  | 0.2 µM |
|  |  |  |  |  |  |  |
| *P. aeruginosa* | rplL | Forward / SYBR | TGGCTCTGACCAACGAAGACATC | 121 | 62°C | 0.3 µM |
|  |  | Reverse / SYBR | GCAACGGTAGCGGCAGCAG |  |  | 0.3 µM |
|  |  |  |  |  |  |  |
| *P. aeruginosa* | rplM | Forward / SYBR | ATGCTGCCGAAGAACCCTCTG | 90 | 62°C | 0.2 µM |
|  |  | Reverse / SYBR | GGGCTGCTGAGCGGTGTG |  |  | 0.2 µM |
|  |  |  |  |  |  |  |
| *P. aeruginosa* | rplN | Forward / SYBR | GGTCGTTCGCACCAAGCAC | 149 | 62°C | 0.5 µM |
|  |  | Reverse / SYBR | ACTTCTCGGTACGGAGTTCACG |  |  | 0.5 µM |
|  |  |  |  |  |  |  |
| *P. aeruginosa* | rplO | Forward / SYBR | GCCGTGGTCACAAAGGTCTG | 148 | 64°C | 0.3 µM |
|  |  | Reverse / SYBR | TCGGAGGTGCGTACTTCAGC |  |  | 0.3 µM |
|  |  |  |  |  |  |  |
| *P. aeruginosa* | rplP | Forward / SYBR | CGAAGTTCCGCAAACAAATGACC | 123 | 62°C | 0.5 µM |
|  |  | Reverse / SYBR | CGATCTGACGAGCAGTAAGGC |  |  | 0.5 µM |
|  |  |  |  |  |  |  |
| *P. aeruginosa* | rplP | Forward / Probe | CCGCCTTACTGCTCGTCA | 148 | 60°C | 0.5 µM |
|  |  | Reverse / Probe | CCCACCCTTACCTTTACCC |  |  | 0.5 µM |
|  |  | Probe | AAGATCTGGATCCGCGTATTCCCTGAC |  |  | 0.2 µM |
|  |  |  |  |  |  |  |
| *P. aeruginosa* | rplQ | Forward / SYBR | TGTTCAACGACCTGGGCAAGC | 141 | 62°C | 0.3 µM |
|  |  | Reverse / SYBR | CTACGACTTCACCGCCGACAG |  |  | 0.3 µM |
|  |  |  |  |  |  |  |
| *P. aeruginosa* | rplR | Forward / SYBR | GCGTCAAGAAAGAAACCCGTCTG | 110 | 62°C | 0.5 µM |
|  |  | Reverse / SYBR | GGCGTAAATGTGCTGGGAAGAG |  |  | 0.5 µM |
|  |  |  |  |  |  |  |
| *P. aeruginosa* | rplS | Forward / SYBR | CCAGGTCAAGGTGAAGGAAGGAG | 124 | 62°C | 0.3 µM |
|  |  | Reverse / SYBR | GCCCACGCCGTTGGAGATC |  |  | 0.3 µM |
|  |  |  |  |  |  |  |
| *P. aeruginosa* | rplV | Forward / SYBR | CGCCGATGTGGATGACCTGAAG | 136 | 62°C | 0.5 µM |
|  |  | Reverse / SYBR | CGCAACCTTGACCGTGATATGG |  |  | 0.5 µM |
|  |  |  |  |  |  |  |
| *P. aeruginosa* | rplV | Forward / Probe | CCGATGTGGATGACCTGAA | 126 | 60°C | 0.5 µM |
|  |  | Reverse / Probe | TGACCGTGATATGGCAAGAC |  |  | 0.5 µM |
|  |  | Probe | GTCAACGAAGGTCGTTCGCTCAAGC |  |  | 0.2 µM |
|  |  |  |  |  |  |  |
| *P. aeruginosa* | rpmE | Forward / SYBR | GACATCCATCCGACTTACGAAGC | 126 | 62°C | 0.5 µM |
|  |  | Reverse / SYBR | GGTGTAGAACGGGTGGCATTC |  |  | 0.5 µM |
|  |  |  |  |  |  |  |
| *P. aeruginosa* | rpmE | Forward / Probe | CGGACATCCATCCGACTTAC | 138 | 60°C | 0.5 µM |
|  |  | Reverse / Probe | TCTGCTTGCCGGTGTAGAA |  |  | 0.5 µM |
|  |  | Probe | GAAGCTATCGAAGCTACCTGCAGCTGC |  |  | 0.2 µM |
|  |  |  |  |  |  |  |
| *P. aeruginosa* | rpoB | Forward / SYBR | CGCCCGCACCAACAAGTAC | 159 | 62°C | 0.3 µM |
|  |  | Reverse / SYBR | TCCACCAGTTGACCCTTCTCG |  |  | 0.3 µM |
|  |  |  |  |  |  |  |
| *P. aeruginosa* | rpsC | Forward / SYBR | ACGCAGACCGCAAGAACTATG |  | 62°C | 0.5 µM |
|  |  | Reverse / SYBR | GCACGGGCACACCCATTTG |  |  | 0.5 µM |
|  |  |  |  |  |  |  |
| *P. aeruginosa* | rpsD | Forward / SYBR | GACCGTCAACATCCCTTCCTACC | 100 | 62°C | 0.5 µM |
|  |  | Reverse / SYBR | GAGTTCCAGAGCCTGAGCGATAC |  |  | 0.5 µM |
|  |  |  |  |  |  |  |
| *P. aeruginosa* | rpsD | Forward / Probe | CCGTCAACATCCCTTCCTAC | 95 | 60°C | 0.5 µM |
|  |  | Reverse / Probe | TTCCAGAGCCTGAGCGATAC |  |  | 0.5 µM |
|  |  | Probe | CAAGTGAAGGCCGGTGACGTCGTAG |  |  | 0.2 µM |
|  |  |  |  |  |  |  |
| *P. aeruginosa* | rpsE | Forward / SYBR | ACTGACCGTGGTGGGTGATG | 100 | 62°C | 0.5 µM |
|  |  | Reverse / SYBR | GCGAGCGGCTTCCATTGC |  |  | 0.5 µM |
|  |  |  |  |  |  |  |
| *P. aeruginosa* | rpsG | Forward / SYBR | GCGTGAAGTGCTGGCTGATC | 95 | 62°C | 0.3 µM |
|  |  | Reverse / SYBR | GCTCGGCAACGGCTTTCTTG |  |  | 0.3 µM |
|  |  |  |  |  |  |  |
| *P. aeruginosa* | rpsH | Forward / SYBR | CTGAAGGACGAAGGCTATATTGCG | 156 | 62°C | 0.2 µM |
|  |  | Reverse / SYBR | TTGCTCAACGGATTTGTACTGACG |  |  | 0.2 µM |
|  |  |  |  |  |  |  |
| *P. aeruginosa* | rpsI | Forward / SYBR | GGCAAGATTTCCATCAACAACCG | 141 | 62°C | 0.5 µM |
|  |  | Reverse / SYBR | TACGCCACCGCCAACGAC |  |  | 0.5 µM |
|  |  |  |  |  |  |  |
| *P. aeruginosa* | rpsJ | Forward / SYBR | CGGATTCGGTTGAAGGCTTTTGAC | 116 | 62°C | 0.3 µM |
|  |  | Reverse / SYBR | CGGGTCGGCAGAGGGATTG |  |  | 0.3 µM |
|  |  |  |  |  |  |  |
| *P. aeruginosa* | rpsL | Forward / SYBR | TCCTCGTACATCGGTGGTGAAG | 107 | 62°C | 0.2 µM |
|  |  | Reverse / SYBR | CGCACGGTGTGGTAACGC |  |  | 0.2 µM |
|  |  |  |  |  |  |  |
| *P. aeruginosa* | rpsN | Forward / SYBR | GCTGACGGTAGCCAAGTACGC | 93 | 62°C | 0.5 µM |
|  |  | Reverse / SYBR | TGGGCATTCCAACGCTCTTCC |  |  | 0.5 µM |
|  |  |  |  |  |  |  |
| *P. aeruginosa* | rpsP | Forward / SYBR | CGCCCCTTCTACCACCTGAC | 101 | 62°C | 0.5 µM |
|  |  | Reverse / SYBR | TCGCCACCAGTCGCAACC |  |  | 0.5 µM |
|  |  |  |  |  |  |  |
| *P. aeruginosa* | rpsQ | Forward / SYBR | GGACAAGACCGTCACCGTACTG | 114 | 62°C | 0.3 µM |
|  |  | Reverse / SYBR | ATGCGGCACTGATTGGATTCG |  |  | 0.3 µM |
|  |  |  |  |  |  |  |
| *P. aeruginosa* | rpsR | Forward / SYBR | AGGATCTCAACACCCTGAAGGC | 93 | 62°C | 0.3 µM |
|  |  | Reverse / SYBR | GCTGACGCTGGTACTTGGC |  |  | 0.3 µM |
|  |  |  |  |  |  |  |
| *P. aeruginosa* | sodB | Forward / SYBR | AGCCTCGAAGAGATCGTCA | 166 | 62°C | 0.5 µM |
|  |  | Reverse / SYBR | ACTTGTCGAAGGAGCCGAAG |  |  | 0.5 µM |
|  |  |  |  |  |  |  |
| *P. aeruginosa* | tsf | Forward / SYBR | TCGAAGTCAACTCCCAGACC | 142 | 62°C | 0.3 µM |
|  |  | Reverse / SYBR | GCCAGACGAGCTTCCTCAC |  |  | 0.3 µM |
|  |  |  |  |  |  |  |
| *P. aeruginosa* | trmD | Forward / SYBR | TGGGCGTCGTCAGCATCTTTC | 130 | 62°C | 0.5 µM |
|  |  | Reverse / SYBR | TGGCGGTCCTCGGTGTAGTC |  |  | 0.5 µM |
|  |  |  |  |  |  |  |
| *P. aeruginosa* | tufB | Forward / SYBR | TCTGCTGAACACCTACGACTTCC | 91 | 62°C | 0.3 µM |
|  |  | Reverse / SYBR | GCCGTTGTCATCCTTGCCTTC |  |  | 0.3 µM |
|  |  |  |  |  |  |  |
| *P. aeruginosa* | 16S rRNA | Forward / SYBR | GGTGGTTCAGCAAGTTGGATGTG | 159 | 62°C | 0.5 µM |
|  |  | Reverse / SYBR | CCAGGTGGTCGCCTTCGC |  |  | 0.5 µM |
|  |  |  |  |  |  |  |
| *P. aeruginosa* | 16S rRNA | Forward / Probe | GCGTAGGTGGTTCAGCAAGT | 120 | 60°C | 0.5 µM |
|  |  | Reverse / Probe | CATTTCACCGCTACACAGGA |  |  | 0.5 µM |
|  |  | Probe | ACTGAGCTAGAGTACGGTAGAGGGTGGTGG |  |  | 0.2 µM |
|  |  |  |  |  |  |  |
| *P. aeruginosa* | 23S rRNA | Forward / SYBR | AGCGACTTATATTCAGTGGCAAGC | 149 | 62°C | 0.5 µM |
|  |  | Reverse / SYBR | TCCTCCAGTCAGTGTTACCTAACC |  |  | 0.5 µM |
|  |  |  |  |  |  |  |
| *P. aeruginosa* | 23S rRNA | Forward / Probe | GGGCTCAAACCACACACC | 90 | 60°C | 0.5 µM |
|  |  | Reverse / Probe | GCTTCTCAACTCACCTTCACAG |  |  | 0.5 µM |
|  |  | Probe | GTAAGTGACGCGGTAGAGGAGCGTTCTGTA |  |  | 0.2 µM |
|  |  |  |  |  |  |  |
| *E. coli* | groEL | Forward / SYBR | ACCGTGGCTACCTGTCTCCT | 188 | 60°C | 0.3 µM |
|  |  | Reverse / SYBR | CGCTTCGCCTTCTACATCTT |  |  | 0.3 µM |
|  |  |  |  |  |  |  |
| *E. coli* | rplE | Forward / SYBR | GAACGCATGTGGGAGTTCTT | 95 | 62°C | 0.3 µM |
|  |  | Reverse / SYBR | CGACCGTCGAAAGACTTAGC |  |  | 0.3 µM |
|  |  |  |  |  |  |  |
| *E. coli* | rplP | Forward / SYBR | TGCTATGACCCGTGCAGTTA | 134 | 62°C | 0.2 µM |
|  |  | Reverse / SYBR | TCAAGGCAACCCAATACTCC |  |  | 0.2 µM |
|  |  |  |  |  |  |  |
| *E. coli* | rplV | Forward / SYBR | CCTGATTCGCGGTAAGAAAG | 120 | 62°C | 0.5 µM |
|  |  | Reverse / SYBR | TCGTTGTGTTCAGCGTTAGC |  |  | 0.5 µM |
|  |  |  |  |  |  |  |
| *E. coli* | rpsD | Forward / SYBR | AGGGCACCGACTTATTCCTT | 136 | 62°C | 0.5 µM |
|  |  | Reverse / SYBR | TTTTGCTTTTCACGCAACTG |  |  | 0.5 µM |
|  |  |  |  |  |  |  |
| *E. coli* | rpmE | Forward / SYBR | TCACCCGAAATACGAAGAAA | 137 | 62°C | 0.3 µM |
|  |  | Reverse / SYBR | CATCACGCTGTTTGCCAGT |  |  | 0.3 µM |
|  |  |  |  |  |  |  |
| *E. coli* | 16S rRNA | Forward / SYBR | TGTAGCGGTGAAATGCGTAG | 159 | 64°C | 0.3 µM |
|  |  | Reverse / SYBR | AGGGCACAACCTCCAAGTC |  |  | 0.3 µM |
| **^a^** Final concentration used for PCR reaction | | | | | | |

*Escherichia coli*

*Pseudomonas aeruginosa*
